# Inference of Convergent Gene Acquisition Among *Pseudomonas syringae* Strains Isolated From Watermelon, Cantaloupe, and Squash

**DOI:** 10.3389/fmicb.2019.00270

**Published:** 2019-02-19

**Authors:** Eric A. Newberry, Mohamed Ebrahim, Sujan Timilsina, Nevena Zlatković, Aleksa Obradović, Carolee T. Bull, Erica M. Goss, Jose C. Huguet-Tapia, Mathews L. Paret, Jeffrey B. Jones, Neha Potnis

**Affiliations:** ^1^Department of Entomology and Plant Pathology, Auburn University, Auburn, AL, United States; ^2^Department of Plant Pathology, North Florida Research and Education Center, University of Florida, Quincy, FL, United States; ^3^Department of Plant Pathology, University of Florida, Gainesville, FL, United States; ^4^Department of Plant Pathology, Faculty of Agriculture, Ain Shams University, Cairo, Egypt; ^5^Faculty of Agriculture, University of Belgrade, Belgrade, Serbia; ^6^Department of Plant Pathology and Environmental Microbiology, Pennsylvania State University, State College, PA, United States; ^7^Emerging Pathogens Institute, University of Florida, Gainesville, FL, United States

**Keywords:** horizontal gene transfer, homologous recombination, pathogen emergence, *Pseudomonas syringae sensu stricto*, cucurbits

## Abstract

*Pseudomonas syringae sensu stricto* (phylogroup 2; referred to as *P. syringae*) consists of an environmentally ubiquitous bacterial population associated with diseases of numerous plant species. Recent studies using multilocus sequence analysis have indicated the clonal expansion of several *P. syringae* lineages, located in phylogroups 2a and 2b, in association with outbreaks of bacterial spot disease of watermelon, cantaloupe, and squash in the United States. To investigate the evolutionary processes that led to the emergence of these epidemic lineages, we sequenced the genomes of six *P. syringae* strains that were isolated from cucurbits grown in the United States, Europe, and China over a period of more than a decade, as well as eight strains that were isolated from watermelon and squash grown in six different Florida counties during the 2013 and 2014 seasons. These data were subjected to comparative analyses along with 42 previously sequenced genomes of *P. syringae* stains collected from diverse plant species and environments available from GenBank. Maximum likelihood reconstruction of the *P. syringae* core genome revealed the presence of a hybrid phylogenetic group, comprised of cucurbit strains collected in Florida, Italy, Serbia, and France, which emerged through genome-wide homologous recombination between phylogroups 2a and 2b. Functional analysis of the recombinant core genome showed that pathways involved in the ATP-dependent transport and metabolism of amino acids, bacterial motility, and secretion systems were enriched for recombination. A survey of described virulence factors indicated the convergent acquisition of several accessory type 3 secreted effectors (T3SEs) among phylogenetically distinct lineages through integrative and conjugative element and plasmid loci. Finally, pathogenicity assays on watermelon and squash showed qualitative differences in virulence between strains of the same clonal lineage, which correlated with T3SEs acquired through various mechanisms of horizontal gene transfer (HGT). This study provides novel insights into the interplay of homologous recombination and HGT toward pathogen emergence and highlights the dynamic nature of *P. syringae sensu lato* genomes.

## Introduction

The Gram-negative bacterial species, *Pseudomonas syringae sensu lato* (in the largest sense), embodies both a pathogenic and phylogenetic complex of strains, which are responsible for numerous plant diseases of economic importance worldwide. Because many of the phytopathogenic bacteria found within this species complex could not be differentiated using traditional phenotypic and biochemical tests, they were classified into distinct pathogenic populations (i.e., pathovars) as defined by their host specificity (Dye et al., 1980). Currently, over 50 pathovars have been described within the seven named species and one genomospecies in *P. syringae sensu lato* (Gardan et al., 1999). These can be distinguished by multilocus sequence analysis (MLSA; Hwang et al., 2005; Young, 2010; Bull et al., 2011; Berge et al., 2014) and whole genome sequence analysis (Marcelletti and Scortichini, 2014; Nowell et al., 2014; Gomila et al., 2017) into phylogroups which correspond to distinct species.

Aside from its role as a plant pathogen, *P. syringae sensu lato* is common in a variety of habitats outside of the agricultural context, including in precipitation, water, soil, and wild plants as a facultative saprophyte (Hirano and Upper, 2000; Morris et al., 2013). Given the ubiquitous nature of this bacterial species, it is not surprising to note that *P. syringae sensu lato* may exhibit a variety of interactions with plants ranging from commensal leaf inhabitant, to opportunistic, and host-specialized phytopathogen. Similarly, some *P. syringae sensu lato* lineages have evolved differing modes of transmission to plants, including via seed and water, which may be reflected in their ecology, metabolic versatility, and other forms of microbial physiology (Baltrus et al., 2017). Several well characterized plant diseases such as bacterial speck of tomato, bleeding canker of European horse chestnut, or bacterial canker of kiwifruit were each linked to the expansion of a genetically monomorphic pathogen lineage (Green et al., 2010; Cai et al., 2011a; McCann et al., 2013). In some cases, the clonal lineages associated with these diseases were closely related to strains collected from environmental sources that were less virulent and had a broader host range than their host-specialized relatives (Cai et al., 2011b; Monteil et al., 2013). This observation has led to the hypothesis that *P. syringae sensu lato* displays an epidemic population structure, whereby novel pathogen lineages emerge from recombining ancestral populations through the acquisition of genes or alleles that provide an adaptive benefit (Vinatzer et al., 2014). Consistent with this hypothesis, gene content fluctuation occurs at an over 100-fold greater rate than amino acid sequence divergence in *P. syringae sensu lato* genomes (Nowell et al., 2014).

Among the various species found within *P. syringae sensu lato*, *P. syringae sensu stricto* (phylogroup 2; referred to as *P. syringae* in the rest of the manuscript) possesses many traits that are characteristic of the species complex as a whole. The strains described here are commonly recovered from environmental sources, maintain large epiphytic populations, are active ice-nucleators, and cause disease on a wide range of plant species (Canfield et al., 1986; Morris et al., 2008; Berge et al., 2014). A distinguishing feature of this group is the production of the phytotoxins syringomycin, syringopeptin, and syringolin, which are virulence factors that exhibit antimycotic activity and facilitate host colonization (Scholz-Schroeder et al., 2001; Misas-Villamil et al., 2013; Nowell et al., 2016). Although a number of agriculturally relevant pathovars have been described within *P. syringae* (Bull and Koike, 2015), strains are commonly identified as *P. syringae* pv. *syringae* based on the detection of genes associated with the biosynthesis of syringomycin (Little et al., 1998; Sorensen et al., 1998; Bultreys and Gheysen, 1999). *P. syringae* pv. *syringae*, which was named for its original host of isolation (*Syringae vulgaris*), has been recorded as a pathogen of over 40 different plant species and has a host range distinct, but overlapping many of the other pathovars found within the same phylogenetic group (Young, 2010). As a result, it is unclear to what degree many of the plant pathogenic bacteria described here exhibit host-specificity and/or represent ecologically separate populations.

Bacterial leaf spot of watermelon (*Citrullus lanatus*), cantaloupe (*Cucumis melo*), and squash (*Cucurbita pepo*) is a common early spring disease that has a worldwide distribution and can cause significant economic losses under cool, wet environmental conditions (Morris et al., 2000; Riffaud et al., 2003; Newberry et al., 2017). The disease was recently recognized as a seedborne disorder of squash (Manceau et al., 2011); however, its etiology in various cucurbit species is likely to have multiple sources (Monteil et al., 2016). Recently, we characterized the *P. syringae* population responsible for bacterial leaf spot epidemics that occurred in commercial production fields of watermelon and squash throughout Florida. Analysis of the population structure indicated that this newly emerging disease was primarily associated with the expansion of a clonal *P. syringae* lineage throughout the state, that was most closely related to the *P. syringae* pv. *syringae* type/pathotype strain, LMG 1247^PT^ (=ICMP 3023^T^), within phylogroup 2b. Additionally, we identified two other clonal lineages collected from either the same, or previous bacterial leaf spot epidemics in the United States that shared a recent a common ancestor with the aforementioned epidemic clone; however, were located in a separate phylogroup within the same species, namely phylogroup 2a (Newberry et al., 2016, 2018). Although, we were able to precisely classify these pathogens within the phylogenetic structure of *P. syringae sensu lato* using MLSA, we were unable to delineate them from other strains collected from a diverse group of plant species or attribute them to any pathovar previously associated with cucurbit hosts, other than *P. syringae* pv. *syringae*.

Here, we investigated the evolutionary processes that led to the emergence of these similar, yet distinct *P. syringae* lineages as successful pathogens of watermelon, cantaloupe, and squash, as well as the genetic factors that distinguish them from other members of this environmentally ubiquitous bacterial population. We obtained high-quality draft genomes for 11 *P. syringae* strains collected from bacterial leaf spot epidemics in the United States, as well as for three strains isolated from symptomatic squash grown in Italy, Serbia, and China over various years. In order to investigate these strains in the context of the larger diversity of *P. syringae*, we analyzed these data together with the genomes of 42 additional *P. syringae* strains collected from diverse plant species and environments available from public sequence databases. We examined the population structure and analyzed patterns of homologous recombination within *P. syringae*. The distribution of previously described virulence factors including type 3 secreted effectors (T3SEs), phytotoxins, and other biologically relevant features were computationally surveyed. Finally, pan-genome association analysis was carried out to identify orthologous groups potentially involved in niche adaptation of the cucurbit lineages. The combined results of this study demonstrate the presence of a hybrid *P. syringae* clone associated with watermelon, cantaloupe, and squash in the United States and Europe and provide evidence for the convergent adaptation of two phylogenetically distinct *P. syringae* populations to cucurbit hosts.

## Materials and Methods

### Bacterial Strains and Sequencing

We selected 14 *P. syringae* strains that were isolated from the symptomatic tissue of watermelon, cantaloupe, and squash over the period of numerous years for shotgun sequencing, as detailed below. Most of these strains were described previously and altogether, comprised three different multilocus haplotypes located in phylogroups 2a and 2b of *P. syringae* (Table 1). Eight strains collected from bacterial leaf spot epidemics that occurred in Florida between 2013 and 2014 were sequenced. These strains were isolated from watermelon and squash grown in six different Florida counties during these epidemics and comprised haplotypes one and two (Newberry et al., 2018). Three strains included for sequencing comprised haplotype three and were isolated from cantaloupe and squash grown in Florida, Georgia, and California between 2000 and 2006 (Newberry et al., 2016). Finally, three additional strains that were isolated from symptomatic squash grown in Italy, Serbia, and China between 2005 and 2013 were also sequenced because they were found to be identical at four partial housekeeping gene sequences to one of the previously mentioned haplotypes (Hwang et al., 2005).

**Table 1:** Draft genome sequencing and assembly statistics for *P. syringae* strains isolated from watermelon, cantaloupe, and squash.

Strain	Origin	Host^a^	Year	MLST^b^	Phylogroup	Contigs (N)	N50 (Kb)	Genome length (Mb)	Accession
13-C2	Florida	WM	2013	1	2b	83	178	5.92	MUHO00000000
13-140A	Florida	WM	2013	1	2b	90	131	5.91	MUHL00000000
13-509A	Florida	SQ	2013	1	2b	107	111	5.89	MUHP00000000
13-139B	Florida	WM	2013	2	2a	83	131	6.37	MVAT00000000
13-429	Florida	WM	2013	2	2a	90	225	6.25	MVAY00000000
14-410	Florida	WM	2014	1	2b	131	99	5.91	MUHQ00000000
14-32	Florida	WM	2014	1	2b	102	124	5.92	MUHM00000000
14-Gil	Florida	WM	2014	1	2b	195	58	5.91	MVAU00000000
03-19A	Florida	CL	2003	3	2a	51	239	6.14	MUHN00000000
200-1	Georgia	SQ	2000	3	2a	91	104	6.14	MVAZ00000000
BS2121	California	SQ	2006	3	2a	71	112	6.22	MVAV00000000
ZUM3584	Italy	SQ	2005	1	2b	129	108	5.97	MVBA00000000
ZUM3984	China	SQ	2008	3	2a	59	263	6.24	MVAX00000000
PS711	Serbia	SQ	2013	1	2b	90	225	5.89	RQXZ01000000

*^a^WM, watermelon; CL, cantaloupe; SQ, squash. ^b^Multilocus sequence type.*

Bacterial strains were purified from single colonies and cultured overnight in nutrient broth. Genomic DNA was extracted using the CTAB-NaCl method (Ausubel et al., 1994), checked for quality using a NanoDrop 2000 (Thermo Scientific, Waltham, MA, United States) and gel electrophoresis, then quantified using a Qubit 3.0 fluorometer (Thermo Fischer, Waltham, MA, United States). Genomic libraries were prepared using a Nextera library preparation kit (Illumina Inc., San Diego, CA, United States) and the DNA was sequenced using the Illumina MiSeq platform at the Interdisciplinary Center for Biotechnology Research, University of Florida. The raw sequence data were subjected to adapter and quality trimming with Scythe^1^ and SolexaQA, respectively (Cox et al., 2010). The quality-trimmed reads were then *de novo* assembled into contigs using the SPAdes Genome assembler (v3.5.0) with the “-careful” option to reduce mismatches in the assembly (Bankevich et al., 2012). The draft genome assemblies were submitted to the Prokaryotic Genomes Automatic Annotation Pipeline (PGAAP) pipeline and the Joint Genome Institute (IMG-JGI) server for annotation (Markowitz et al., 2012; Tatusova et al., 2016). The sequencing, assembly statistics, and collection information are presented in Table 1.

### Pan-Genome Association Analysis

The strains sequenced in this study were investigated in context of *P. syringae* by including 42 additional genomes that were publicly available from the National Center for Biotechnology Information (NCBI) GenBank for analysis (Table 2). These genomes were selected to represent the diversity of phytopathogenic bacteria previously described within the species and are distributed among phylogroups 2a, 2b, and 2d (Berge et al., 2014; Bull and Koike, 2015). Representatives of phylogroup 2c, otherwise known as *P. congelans*, were not included in this analysis as they are not known to be phytopathogenic (Mohr et al., 2008). The genome assemblies were re-annotated with Prokka to generate GFF3 files (Seemann, 2014), which were then used as input for Roary pan-genome pipeline (v.3.6.1, Page et al., 2015). Orthologous groups were clustered using the CD-Hit and MCL algorithms with a BLASTp cut-off set to 95% along with the “-s” option to prevent the splitting of orthologous groups containing paralogs. The output of Roary was further analyzed using Scoary to test for associations between the presence/absence of orthologous groups and cucurbit-associated lineages (Brynildsrud et al., 2016). The population structure (as described below) was used to control for spurious associations in the estimation of probabilities and orthologous groups with a Bonferroni *p* ≤ 10^−5^ were reported.

**Table 2:** List of genomes included in comparative analyses including pathovar classification, host of isolation, and phylogenetic classification based on MLSA.

Strain	Pathovar	Isolation source	Phylogroup	Accession	Reference
Alf3	*syringae*	*Medicago sativa* (Alfalfa)	2b	JPNN00000000.1	Harrison et al., 2016
BS0292	*aptata*	*Beta vulgaris* (Sugar beet)	2b	FOVV00000000.1	–
BS3827	*aptata*	*Beta vulgaris* (Sugar beet)	2b	FOQB00000000.1	–
BS3829	*aptata*	*Beta vulgaris* (Sugar beet)	2b	FOPR00000000.1	–
CC457	NA	*Cucumis melo* (Cantaloupe)	2b	AVEB00000000.2	Baltrus et al., 2014b
HS191	*syringae*	*Panicum miliaceum* (Millet)	2b	NZ_CP006256.1	Ravindran et al., 2015
ICMP459^PT^	*aptata*	*Beta vulgaris* (Sugar beet)	2b	LJRP00000000.1	Thakur et al., 2016
PP1	*pisi*	*Pisum sativum* L. (Pea)	2b	AUZR00000000.2	Baltrus et al., 2014a
Pav013	*avellanae*	*Corylus avellana* (European hazelnut)	2b	GCA_000302795.1	O’Brien et al., 2012
Pav037	*avellanae*	*Corylus avellana* (European hazelnut)	2b	GCA_000302815.1	O’Brien et al., 2012
2507	*syringae*	*Triticum aestivum* (Wheat)	2b	LYUO00000000.1	Sultanov et al., 2016
41A	*syringae*	*Prunus armeniaca* (Armenian plum)	2b	JYHJ00000000.1	Bartoli et al., 2015
1845	*syringae*	*Helianthus* L. (Sun flower)	2b	LYUP00000000.1	Sultanov et al., 2016
B64	*syringae*	*Triticum aestivum* (Wheat)	2b	ANZF00000000.1	Dudnik and Dudler, 2013
CRAFRU11	*syringae*	*Corylus avellana* (European hazelnut)	2b	ATSU00000000.1	Scortichini et al., 2013
CRAFRU12	*syringae*	*Corylus avellana* (European hazelnut)	2b	ATSV00000000.1	Scortichini et al., 2013
ICMP11168	*syringae*	*A. deliciosa* (Kiwifruit)	2b	LKGV00000000.1	Visnovsky et al., 2016
ICMP3023^T^	*syringae*	*Syringa vulgaris* (Lilac)	2b	LJRK00000000.1	Thakur et al., 2016
ICMP3947^PT^	*lapsa*	*Triticum aestivum* (Wheat)	2b	LJQQ00000000.1	Thakur et al., 2016
ICMP4394^PT^	*atrofaciens*	*Triticum aestivum* (Wheat)	2b	LJPO00000000.1	Thakur et al., 2016
MB03	NA	*Populus lasiocarpa* (Poplar)	2b	LAGV00000000.1	–
NCPPB4273^T^	*coryli*	*Corylus avellana* (European hazelnut)	2b	AWQP00000000.1	Marcelletti and Scortichini, 2014
SM	*syringae*	*Triticum aestivum* (Wheat)	2b	APWT00000000.1	Dudnik and Dudler, 2013
BRIP34881	NA	*Hordeum vulgare* (Barley)	2b	AMXL00000000.1	Gardiner et al., 2013
A2	*syringae*	*Pyrus calleryana* (Pear)	2a	LGKU00000000.1	Mott et al., 2016
CFBP1754^PT^	*papulans*	*Malus sylvestris* (Apple)	2a	JYHI00000000.1	Bartoli et al., 2015
UMAF0158	*syringae*	*Mangifera indica* (Mango)	2a	NZ_CP005970.1	Martínez-García et al., 2015
31R1	NA	*Zea mays* L. (Corn)	2a	GCA_900105295.1	Lindow, 1987
BRIP39023	NA	*Triticum aestivum* (Wheat)	2a	AMZX00000000.1	Dudnik and Dudler, 2013
ICMP11293	*syringae*	*A. deliciosa* (Kiwifruit)	2a	LKEP00000000.1	Visnovsky et al., 2016
NFACC10-1	NA	*Panicum virgatum* (Switchgrass)	2a	GCF_900119195.1	–
Pc58^T^	NA	*Prunus cerasus* (Sour cherry)	2a	GCA_900074915.1	Kałuz̊na et al., 2016
B301D	*syringae*	*Pyrus communis* (Comice pear)	2d	GCA_000988485.1	Ravindran et al., 2015
B728a	*syringae*	*Phaseolus vulgaris* (Bean)	2d	NC_007005.1	Feil et al., 2005
2339	*syringae*	*Prunus avium* (Sweet cherry)	2d	LIHU00000000.1	Nowell et al., 2016
2340	*syringae*	*Pyrus* sp. (Pear)	2d	LIHT00000000.1	Nowell et al., 2016
HRI-W7872	*syringae*	*Prunus domestica* (Plum)	2d	LIHS00000000.1	Nowell et al., 2016
HRI-W7924	*syringae*	*Prunus cerasus* (Sour cherry)	2d	LIHR00000000.1	Nowell et al., 2016
ICMP13102	*syringae*	*A. deliciosa* (Kiwifruit)	2d	LKEO00000000.1	Visnovsky et al., 2016
PD2774	*syringae*	*A. chinensis* (Kiwifruit)	2d	LKEL00000000.1	Visnovsky et al., 2016
ATCC10853^PT^	*aceris*	*Acer* L. (Maple)	2d	LGAR00000000.1	–
USA011	NA	Freshwater	2d	AVDX00000000.2	Baltrus et al., 2014b

### Analysis of Population Structure and Interlineage Recombination

Initial analysis of the population structure was conducted by calculating the average nucleotide identities between genomes using the MUMmer algorithm (Marçais et al., 2018), with the Python package pyani^2^. Subsequently, a core genome alignment was constructed using the program Parsnp (Treangen et al., 2014). Locally collinear blocks (LCBs) of maximal unique matches shared across all genome assemblies were identified and aligned against the gold standard reference genome of *P. syringae* pv. *syringae* strain B728a (NC_007005.1). Single nucleotide polymorphisms (SNPs) located on LCBs < 200 bp or other regions of poor alignment were removed from the data set to generate a concatenated alignment of high-quality core genome SNPs. This concatenated SNP alignment was used to infer a maximum likelihood phylogeny using iQTree (v.1.6.4) with the Jukes-Cantor model of nucleotide substitution (Nguyen et al., 2015). Branch support was assessed using the ultrafast bootstrap method with 1,000 replicates (Minh et al., 2013) and the phylogenetic tree was visualized and annotated using FigTree (v.1.4.2^3^).

To analyze patterns of homologous recombination within *P. syringae*, the core genome alignment generated with Parsnp was used as input for analysis with fastGEAR using the default settings. The fastGEAR algorithm employs, BAPS, a Bayesian hierarchical clustering method to infer sequence clusters in an alignment (Corander et al., 2003). This was followed by a hidden Markov model (HMM), which was used to collapse clusters that share a common ancestry in at least 50% of the sites into lineages. The program detects “recent” recombination events between lineages using a HMM and the origin of the recombinant sequence is assigned to the lineage with the highest probability at that position. Similarly, “ancestral” recombination events that are shared by all strains which comprise a lineage are identified; however, the origin of these recombination events cannot be inferred due to their conserved nature (Mostowy et al., 2017). The statistical significance of recombination predictions was tested using a Bayes factor (BF) > 1 for recent recombination events and BF > 10 for ancestral recombination events. This analysis was conducted with two alternative genome alignments using completed reference genomes of *P. syringae* pv. *syringae* strains UMAF0158 (NZ_CP005970.1) and HS191 (NZ_CP006256.1) to validate the lineage prediction and proportion of gene flux between lineages. Finally, to examine the evolutionary history of *P. syringae* in the absence of recombination, the positions in the core genome alignment which corresponded to the predicted recent recombination events were removed and SNPs extracted using the program SNP-sites (Page et al., 2016). This recombination-filtered SNP alignment was then used to construct a maximum likelihood phylogeny as described above.

### Functional Analysis of Recent Recombination Events

To characterize the functional impact of the recombinant genes leading to the emergence of phylogroup 2b-a, BLASTn (E-value ≤ 1e^−50^) was used to map the recent recombination events predicted by fastGEAR back to the genome assemblies of three strains (13-140A, ZUM3584, HS191), representative of the different recombination profiles in the dataset. The Clusters of Orthologous Groups (COG) and Kyoto Encyclopedia of Genes and Genomes (KEGG) orthology IDs corresponding to recombinant and non-recombinant genes were subsequently extracted from the IMG annotations and a linear regression analysis was conducted to compare the composition of recombinant vs. non-recombinant core genes across the COG functional categories. Additionally, the KEGG orthology IDs were used to compare the distribution of recombinant genes across the KEGG BRITE functional annotations among the three different recombination profiles.

After noting evidence for extensive recombination affecting the *hrp/hrc* pathogenicity island of phylogroup 2b-a cucurbit strains in the fastGEAR output, amino acid sequence alignments for the 27 open reading frames that comprise *hrp/hrc* gene cluster, as described by Alfano et al. (2000), were constructed using a sub-set of 54 *P. syringae* genomes. Individual gene alignments were subjected to a phylogenetic analysis with FastTree 2 using the gamma time reversible model (Price et al., 2010). A gene was considered to be recombinant among the 2b-a lineages if it clustered into a monophyletic group with other of members of phylogroup 2a, rather than 2b, as predicted by the fastGEAR algorithm. To illustrate these relationships, a phylogenetic network was constructed with a concatenated alignment of the entire *hrp/hrc* gene cluster using the NeighborNet method and p-distance in SplitsTree 4 (Huson and Bryant, 2006).

### Analysis of Type 3 Secreted Effectors (T3SEs) and Phytotoxins

Reference sequences were obtained from the public T3SE database^4^ and used to query the genome assemblies using BLASTn (E-value ≤ 1e^−5^). A given T3SE was considered to be present in a genome if the alignment with the reference sequence displayed at least 80% sequence identity over 80% of the query length. If genomic sequences orthologous to a given reference sequence were split between two contigs, then the sequences were concatenated to determine their presence in the assemblies. For each T3SE, an alignment of the subject sequence with the curated reference sequence was used to record putative frameshift mutations or other disruption of the coding sequence. The distribution of T3SEs was then used to construct a binary matrix, where 1 indicated the presence of a gene, 0.5 indicated a putative pseudogene, and 0 if the gene was absent. Hierarchical clustering of the binary matrix was performed with the Python package, hclust2^5^. A similar strategy described by Baltrus et al. (2011) was used to identify genes involved in the biosynthesis of the following phytotoxins: tabtoxin, phaseolotoxin, mangotoxin, syringomycin, syringolin, and syringopeptin.

### Identification of Plasmids and Genomic Islands

The presence of plasmids was predicted for the strains sequenced in this study using plasmidSPAdes (Antipov et al., 2016). Assembled contigs were screened using Microbial Genome BLAST against the complete plasmid database to identify putative plasmid sequences. These contigs were subsequently screened for T3SEs and other virulence factors as described above. Genomic islands and other signatures of horizontal gene transfer (HGT) were detected using the Island Viewer 4 server (Bertelli and Brinkman, 2018). Genomic islands (GIs) were predicted using the reference strains *P. fluorescens* SBW25, *P. protegens* Pf-5, *P. aeruginosa* PAO1, and *P. syringae pv. syringae* B728a. The predicted GIs were analyzed for gene content and extracted from the genome assemblies to construct multiple sequence alignments using the Mauve software package (Darling et al., 2004).

### Pathogenicity Assays

The pathogenicity of most of the bacterial strains sequenced in this study was examined previously (Newberry et al., 2016, 2018). However, to make direct comparisons with strains collected from independent disease outbreaks, a sub-set of 10 *P. syringae* strains representative of the genetic diversity and geographic source of isolation in the collection were selected for further pathogenicity testing. A strain that was originally isolated from diseased proso millet, *P. syringae* pv. *syringae* HS191 (Gross and DeVay, 1976; Ravindran et al., 2015), was also included in these experiments due to its genetic similarity to the cucurbit strains examined here. Bacterial strains were cultured overnight on King’s medium B agar, suspended in a sterile MgSO_4_^∗^7H_2_O solution (10 mM), and adjusted to ∼1 × 10^8^ colony forming units (CFU ml^−1^) spectrophotometrically (OD_600_ = 0.1). Two-week-old seedlings of watermelon cv. Troubadour (Harris Seeds, Rochester, NY, United States) and squash cv. Conqueror III (Seminis Vegetable Seeds) were sprayed with the bacterial suspension until run-off, incubated in a humidity chamber at 100% RH for 48 h, then placed under growth room conditions at 21°C and ∼70% RH with a 12 h photoperiod. Plants sprayed with the sterile MgSO_4_^∗^7H_2_O solution served as a negative control. Two weeks after inoculation, the total proportion of necrotic/symptomatic leaf tissue was rated visually from 0 to 8 using a modified version of the Horsfall–Barratt scale (Horsfall and Barratt, 1945): 0 = no symptoms, 1 = 1–3%, 2 = 3–6%, 3 = 6–12%, 4 = 12–25%, 5 = 25–50%, 6 = 50–75%, 7 = 75–87%, 8 = 87–100%. Five replicates were included for each strain/host combination, and the experiment was conducted twice. A non-parametric analysis of variance (ANOVA) was used to test for differences among the treatments and all statistical analyses were conducted using JMP Pro 13 (SAS Institute, Cary, NC, United States).

## Results

### Population Structure of *P. syringae sensu stricto*

**FIGURE 1:**
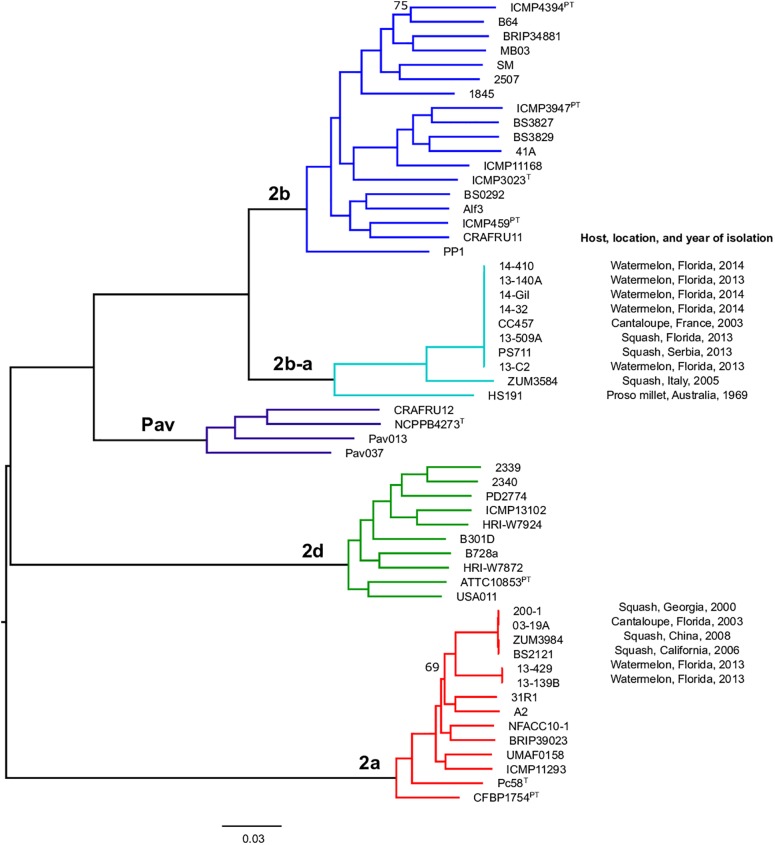
Population structure of *P. syringae sensu stricto*. Mid-point rooted, maximum likelihood phylogeny based on 246,510 SNPs extracted from a core genome alignment of 2.43 Mb. Sequence clusters identified by BAPS analysis are color coded and phylogroup designations are labeled at the nodes. Bootstrap support was ≥98% except where otherwise indicated and the scale bar shows the number of substitutions per site.

Previous studies using MLSA described three phylogroups of phytopathogenic bacteria within *P. syringae sensu stricto* (Berge et al., 2014; Bull and Koike, 2015). Maximum likelihood reconstruction of the *P. syringae* core genome based on 246,510 polymorphic sites extracted from an alignment of 2.43 Mb revealed the presence of two well-supported phylogenetic groups in addition to those delineated through MLSA (2a, 2b, and 2d), designated here as 2b-a and Pav (Figure 1). The between-phylogroup average nucleotide identities (ANI) were primarily equal and ranged from 94.61 to 95.54% ANI. However, phylogroup 2b-a was intermediate of 2b (97.43% ANI), Pav (96.15% ANI), and 2a (96.00% ANI). Similar levels of sequence identity (between 98.16 and 98.98% ANI) were recorded within all *P. syringae* phylogroups (Table 3). Phylogroup 2b contained a number of strains collected from diverse monocot and herbaceous dicot plant species including the type strain, *P. syringae* pv. *syringae* ICMP3023^T^, as well as other previously described pathovars such as *P. syringae* pvs. *aptata* ICMP459^PT^, *atrofaciens* ICMP4394^PT^, *pisi* PP1, and *lapsa* ICMP3947^PT^. Phylogroup 2b-a branched as a sister group to 2b and contained many cucurbit strains that were isolated from plants grown in Florida, Italy, France, and Serbia between 2003 and 2014. All of these strains were of the same clonal lineage, except for ZUM3584, and shared a recent common ancestor with that of *P. syringae* pv. *syringae* HS191, which was isolated in Australia from diseased proso millet in 1969 and was more distantly related. The other novel *P. syringae* phylogroup inferred in this analysis, phylogroup Pav, consisted of strains associated exclusively with hazelnut decline that were classified primarily as *P. syringae* pv. *avellanae* (O’Brien et al., 2012) and branched between phylogroups 2b and 2d. The cucurbit strains located in phylogroup 2a branched from each other into two distinct lineages that were nested among other strains isolated from diverse plants and environments. A well supported split was observed between the clones collected in California and China (BS2121 and ZUM3984) and those in Florida and Georgia (03-19A and 200-1), which differed by less than 300 SNPs in the core genome alignment and represented distinct haplotypes within the lineage (Figure 1).

**Table 3:** Average percent identity within and between *P. syringae* phylogroups^a^.

	2a	2b	Pav	2b-a	2d
2a	98.95				
2b	94.80	98.35			
Pav	95.20	96.55	98.16		
2b-a	96.00	97.43	96.15	98.68	
2d	94.61	95.20	95.54	95.05	98.75

*^a^Percent identity was calculated after correcting for clones in the dataset.*

### Patterns of Homologous Recombination Between *P. syringae sensu stricto* Phylogroups

**FIGURE 2:**
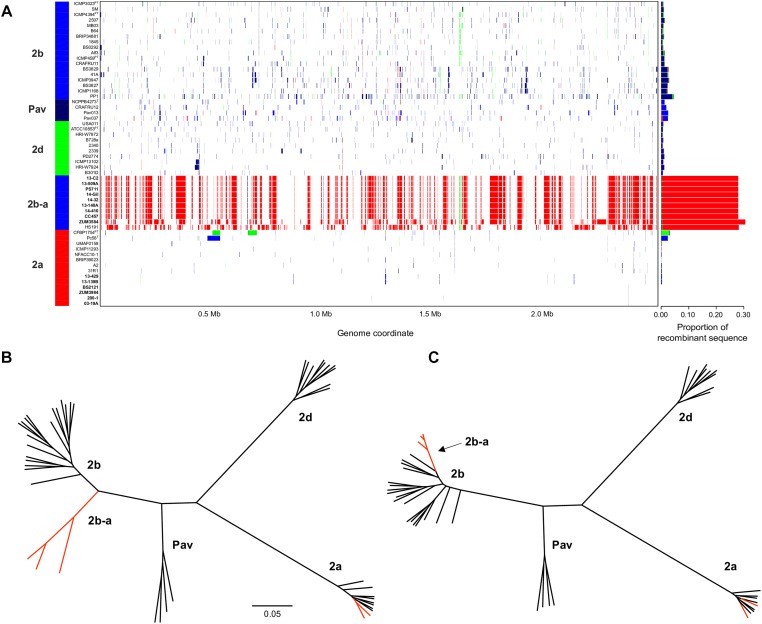
Inference of recent homologous recombination between *P. syringae sensu stricto* phylogroups. Distribution, origin, and proportion of recent recombination events across the *P. syringae* core genome as predicted by fastGEAR. The lineage predictions are color coded and phylogroups labeled accordingly. Strains isolated from cucurbits hosts are shown in bold **(A)**. Unrooted, maximum likelihood phylogeny based on 246,510 core genome SNPs **(B)** and unrooted, maximum likelihood phylogeny based on 81,421 recombination free SNPs **(C)**. Branches corresponding to strains isolated from cucurbit hosts and the millet strain, HS191, are labeled in red. The scale bar indicates the number of substitutions per site.

The sequence clusters identified by the Bayesian statistical clustering method were congruent to the phylogroups supported by the maximum likelihood phylogeny. However, fastGEAR collapsed phylogroups 2b and 2b-a into a single lineage, while the others remained distinct. The average proportion of recent recombination in the core genome of *P. syringae* phylogroups (± the standard deviation) was estimated to be 0.71 ± 1.13% for 2a, 1.44 ± 1.16% for 2b, 2.03 ± 0.06% for Pav, and 0.81 ± 0.20% for 2d. In contrast, the proportion of recent recombination in the core genome of phylogroup 2b-a ranged from 27.98 to 30.54%, with ≥98.69% of the recombinant sequences predicted to have originated from phylogroup 2a. Between 178 and 213 recent recombination events were dispersed across the core genome of 2b-a lineages, where ZUM3584 and HS191 displayed variation in distribution of recent recombination events in relation to the strains collected in Florida, France, and Serbia (Figure 2A). Although evidence of ancestral recombination was minimal or undetected among the 2a (0.49%), 2b (0.00%), and 2d (1.32%) lineages, 40.09% of the phylogroup Pav core genome was acquired through ancestral recombination events (Supplementary Figure S1). After removing 1,592,717 positions affected by recent recombination from the core genome alignment (64% of the original alignment), a second phylogenetic analysis was conducted using 81,421 recombination-free SNPs. Overall, the topology of the recombination filtered phylogeny was congruent to that of the unfiltered tree. However, phylogroup 2b-a appeared as a branch within phylogroup 2b, rather than as a distinct group and with considerably shorter branch lengths as compared to the unfiltered phylogeny (Figure 2B,C).

### Functional Analysis of Recombinant Genes Leading to the Emergence of Phylogroup 2b-a

**FIGURE 3:**
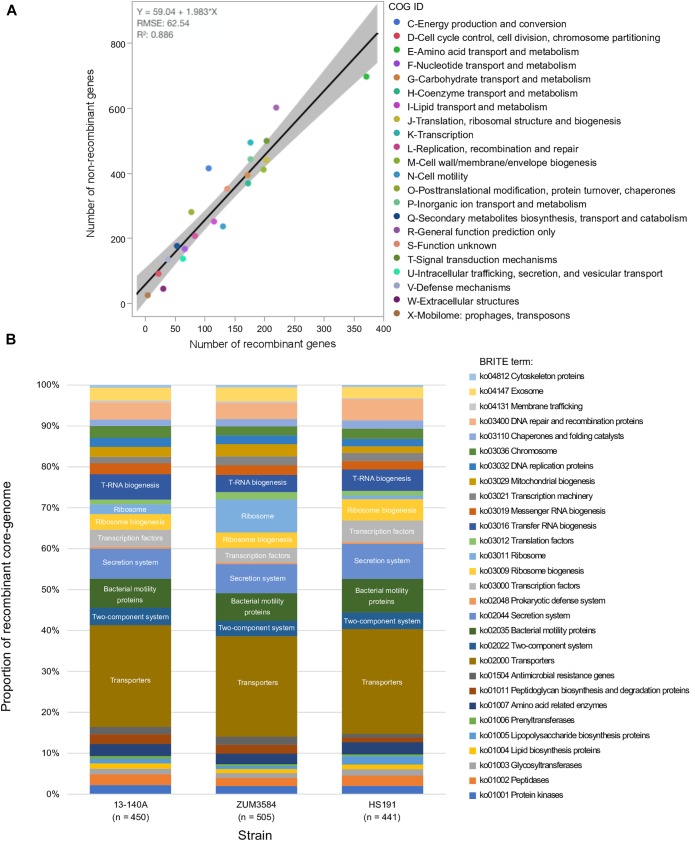
Functional impact of recombination on phylogroup 2b-a. Comparison of COG category compositions corresponding to the recombination-free and recombinant core genome of phylogroup 2b-a. **(A)**. Stacked bar-chart showing the proportion of KEGG BRITE functional categories assigned to the recombinant core genome of three strains representative of phylogroup 2b-a recombination profiles **(B)**.

The recombinant regions predicted by fastGEAR corresponded to 3,094 coding sequences among strains 13-140A, ZUM3584, and HS191, of which 2,812 were assigned to a COG functional category. Likewise, 6,875 of 7,654 coding sequences were assigned to COG categories corresponding to regions of the core genome where no signal of recombination was detected. No significant difference in the effect of COG category on recombination was observed between strains (*P* = 0.15) and a strong linear relationship between recombinant and recombination-free core genes was noted across the general functional groups (*R*^2^ = 0.886; Figure 3A). Although, a Grubb’s test for outliers indicated that no COG category was significantly over- or underrepresented with recombinant genes (data not shown), the top three categories skewed by recombination included amino acid transport and metabolism (E), cell motility (N), and extracellular structures (W). Functional categorization of recombinant core genes utilizing the KEGG BRITE database supported these observations. Approximately 25% of the classifiable genes impacted by recombination were ATP-dependent transporters associated with numerous metabolic pathways, followed by bacterial motility proteins and secretions systems, which each comprised approximately 7–8% of the recombinant genes in each strain (Figure 3B).

**FIGURE 4:**
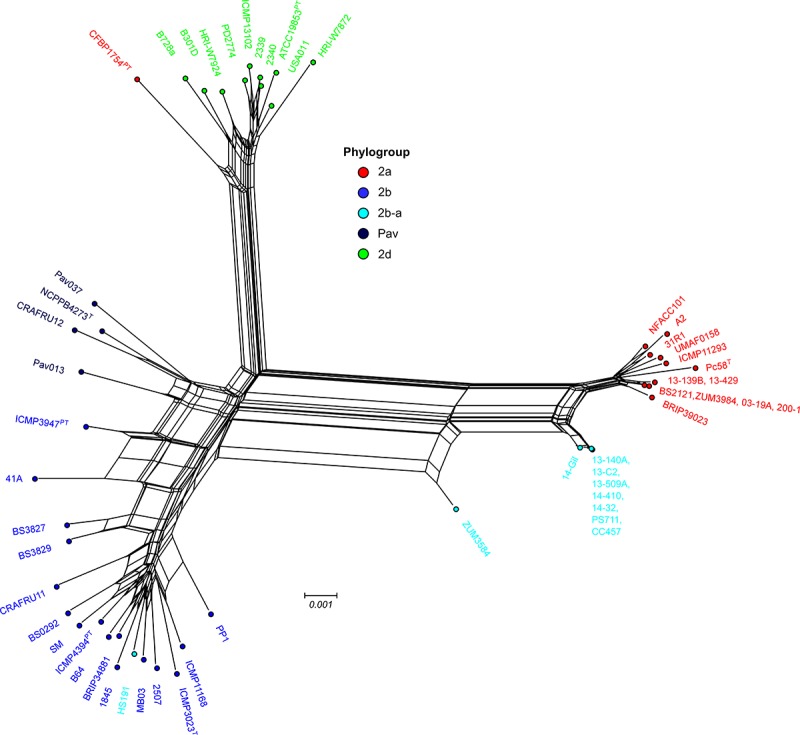
Evolutionary history of the *P. syringae hrp/hrc* pathogenicity island. Phylogenetic network based on a concatenated amino acid alignment of the 27 open reading frames that comprise the *hrp/hrc* gene cluster (7,509 aa) for a sub-set of 54 *P. syringae* genomes. The network was generated using the NeighborNet method and p-distance in Splitstree 4 software. The phylogroup classifications of lineages based on the core-genome (Figure 1) are color coded.

Examination of the specific secretion systems impacted by recombination indicated that many of the genes associated with the biosynthesis of type III secretion system (i.e., *hrp/hrc* gene cluster) were recombinant among strains 13-140A and ZUM3584, whereas no signals of recombination were detected in the *hrp/hrc* cluster of strain HS191. Phylogenetic analyses showed that 17 *hrp/hrc* genes clustered the cucurbit strains isolated in Florida, France, and Serbia into a monophyletic group with other members of phylogroup 2a and were therefore considered recombinant, while strain ZUM3584 carried 11 recombinant alleles (data not shown). A phylogenetic network constructed with a concatenated alignment of the entire *hrp/hrc* cluster (7,509 aa) was largely concordant with that of the core genome phylogeny. This analysis showed the phylogroup 2b-a cucurbit strains branching from other 2a lineages, while ZUM3584 was placed in a hybrid position in the phylogenetic network and HS191 clustered among members of phylogroup 2b (Figure 4).

### Distribution of Type 3 Secreted Effectors (T3SEs) and Phytotoxins

**FIGURE 5:**
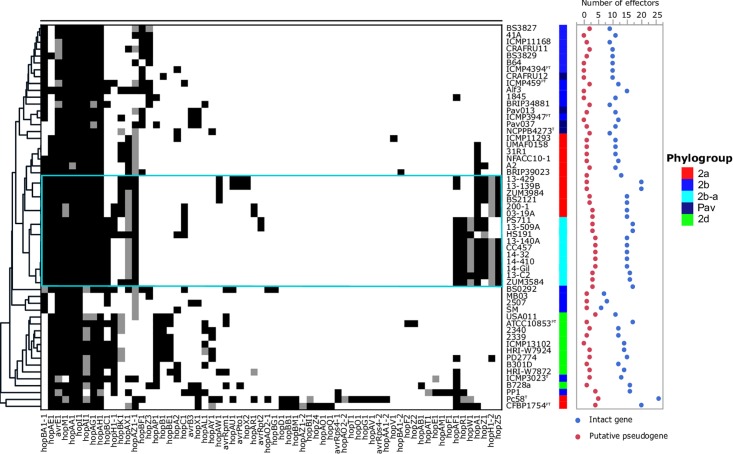
Type 3 secreted effector profiles of *P. syringae sensu stricto*. Black squares indicate the presence of an intact coding sequence and gray squares indicates the presence of a putative pseudogene. The dendrogram was constructed based on the presence/absence of T3SEs using Pearson’s correlation coefficient and the average linkage method with Hclust2. The effector profiles for strains isolated from cucurbit hosts and the millet strain, HS191, are shown inside the cyan box. Phylogroup classifications of lineages based on the core-genome are color coded.

The T3SE repertoires of the 56 genomes analyzed in this study are presented in Figure 5. The cucurbit strains carried between 17 and 21 potentially functional or disrupted effector genes, while an average of 13 T3SEs were present among other *P. syringae* lineages. Hierarchical clustering based on the presence/absence of effector genes revealed a loose correlation between the core genome evolution and effector repertoires, with most members of the primary *P. syringae* phylogroups clustering into distinct groups. The phylogroup 2b-a and 2a cucurbit strains carried similar effector profiles and clustered together along with other 2a lineages. The T3SE *hopA1* was exclusive to most of the strains within this group and was noted to be the only gene present in the exchangeable effector locus of phylogroup 2b-a strains (except for HS191 which carried *hopA2*), along with its corresponding chaperone, *shcA*. The effector *hopZ5* was present exclusively in the genomes of all but two cucurbit strains (13-509 and PS711) within *P. syringae sensu stricto* and displayed 98.27% amino acid sequence identity to the *hopZ5* allele present in the *P. syringae* pv. *actinidiae* biovar 3 of phylogroup 1. This effector was linked to *hopH1*, present with a point deletion, resulting a frameshift mutation in the gene. Several effector genes found to be sparsely distributed across *P. syringae* were also identified among the cucurbit-associated lineages. Most of these genes were present on an integrative and conjugative element (ICE) or plasmid loci (see results below) and included *avrRpt2* in strains 13-509A and PS711; *hopAR1* in strains 200-1 and 03-19A; *avrPto1*, *hopAU1*, and *hopX2* in strains 13-139B and 13-429; and *hopAW1*, which was common to all phylogroup 2a-cucurbit strains except 200-1 and 03-19A.

We also investigated the presence of phytotoxin biosynthetic gene clusters in the genome assemblies, which displayed a simpler distribution. The mangotoxin gene cluster was conserved across *P. syringae*. Evidence for the complete syringomycin, syringolin, and syringopeptin biosynthetic pathways was also present in most *P. syringae* genomes including the cucurbit strains examined here, although was notably absent from strains associated with diseases of woody hosts including all members of phylogroup Pav, *P. cerasi* 58^T^, and *P. syringae* pv. *papulans* CFBP1754^PT^. Additionally, phylogroup 2a cucurbit strains 03-19A, 200-1, BS2121, and ZUM3984 carried *dcd2*, which involved in phaseolotoxin biosynthesis, but lacked other components of this biosynthetic pathway (Supplementary Figure S2). Ravindran et al. (2015) previously noted that phylogroup 2b-a strain HS191 was negative for ice-nucleation activity and carried an ∼2 Kb truncation in the center of the ice-nucleation protein, *inaZ*. As the 2b-a cucurbit strains were previously found to be negative for ice-nucleation activity (Newberry et al., 2018), we investigated the presence of *inaZ* in the genome assemblies of these strains and found the same truncation (data not shown).

### Pan-Genome Association Analysis

We investigated the pan-genome of *P. syringae* to identify orthologous groups (OGs) associated the cucurbit strains and therefore, potentially involved in niche adaptation. Among the 19,613 OGs that comprised the *P. syringae* pan-genome, only seven were significantly associated with both phylogroup 2a and 2b-a cucurbit strains (Bonferroni *p* ≤ 10^−5^). Among these, two displayed 100% specificity, indicating that these OGs were completely absent from the genomes of related *P. syringae* strains and included the T3SE *hopZ5* and a hypothetical protein also present in the ICE locus. Several other OGs with known functions in virulence displayed significant associations and included the T3SE *hopA1*, its corresponding chaperone *shcA*, and the type VI secreted effector *vgrG*. The gene that displayed the strongest association with the cucurbit lineages was a hypothetical protein of 63 aa in length, adjacent to a putative iron-sulfur binding gene cluster (Table 4).

**Table 4:** Top orthologous groups associated with phylogroup 2b-a and 2a cucurbit lineages.

Locus tag^a^	Gene/Annotation^b^	Sensitivity^c^	Specificity^c^	Bonferroni (*p*)
106332	Hypothetical protein	100	95.12	1.36E^−07^
1061120	Hypothetical protein (ICE)	86.67	100	9.03E^−07^
106173	*hopZ*5 (ICE)	86.67	100	9.03E^−07^
1099230	*shcA*	100	87.80	1.55E^−05^
1056171	Hypothetical protein	93.33	92.68	2.67E^−05^
107291	*vgrG*	100	86.37	5.43E^−05^
1099229	*hopA*1	100	86.37	5.43E^−05^

*^a^IMG locus tags reported for strain 13-140A, the identifier Ga0170668_precedes each locus tag number. ^b^ICE shown in parentheses indicates that a gene was present in the integrative and conjugative element locus. ^c^Sensitivity indicates the proportion of the target population (cucurbit lineages) in which an orthologous group was present, and specificity shows the proportion of the non-target population in which an orthologous group was absent.*

### Convergent Acquisition of T3SEs and Other Putative Virulence Factors Through Integrative and Conjugative Elements and Plasmids

**FIGURE 6:**
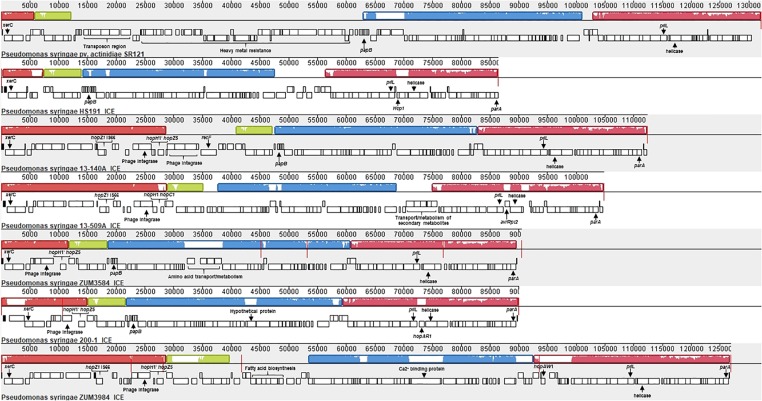
Mauve alignment of the integrative and conjugative elements from *P. syringae* pv. *actinidiae* SR121 (accession no. KX009066), *P. syringae* pv. *syringae* HS191 and representative cucurbit strains from phylogroups 2a and 2b-a. Colored blocks represent locally co-linear blocks without rearrangements and red lines show contig breaks. The histogram inside each box shows the average level of conservation in that region of the genome sequence. Areas that are completely white were not aligned and contain sequence elements specific to a particular ICE. Gene annotations and general functional annotations are labeled where available.

Analysis with Island Viewer 4 revealed that all cucurbit strains carried a predicted genomic island between approximately 90 and 125 Kb in size that was similar in structure to an ICE described in *P. syringae* pvs. *actinidiae* and *phaseolicola* of phylogroups 1 and 3, respectively (Pitman et al., 2005; McCann et al., 2013). The boundaries of the island were delineated by *parA* and *xerC* at opposite ends, each flanking a repeat region overlapping *tRNA_lys_*, which serve as the attachment sites for the island (Pitman et al., 2005). The contigs corresponding to predicted ICE loci were subsequently extracted from the genomes of representative cucurbit strains (divided between one and five contigs per genome) and used to construct a multiple sequence alignment along with the complete ICE sequences of *P. syringae* pv. *actinidiae* (Psa) SR121 (Accession no. KX009066) and *P. syringae* pv. *syringae* HS191 (Figure 6). We were unable to confirm whether the predicted ICE carried by strains 13-429 and 13-139B was intact due to the presence of the pyoverdine biosynthetic gene cluster (∼60 Kb) in the center of the ICE region of these two strains and therefore, were not included in the alignment.

This analysis revealed that the putative ICEs displayed a mosaic structure characterized by differing mobile genetic elements and gene cassettes. It also revealed the overall synteny of the ICEs was conserved and that the core ICE genes displayed a high degree of homology (ranging from 96 to 99% average pairwise identity) with the ICE of Psa strain SR121. Similar to the ICE described in *P. syringae* pv. *phaseolicola* strain 1302A (Pitman et al., 2005), *hopAR1* was carried in the ICE of strain 200-1 between a predicted helicase and *pilL*, while *avrRpt2* was found at the same locus in strain 13-509A and HS191 carried a predicted type VI secretion protein (*hcp1*). Similarly, *hopZ5* and a disrupted copy of *hopH1* was present in the ICEs of all cucurbit strains except 13-509A, which carried *hopC1* and an intact copy of *hopH1* at the same locus. Both of these effector pairs were flanked by a predicted phage integrase and site-specific recombinase (*xerD*) that shared 97.05 and 93.13 aa identity, respectively.

The effector *hopZ1* was also present in the ICE of all but two cucurbit strains (ZUM3584, 200-1) and was located approximately 10 Kb downstream of *hopZ5*/*hopC1*, near the boundary of the island. Interestingly, *hopZ1* was also carried by strain HS191 and was linked to a similar integrase/recombinase gene cassette. However, in HS191, *hopZ1* was present in the exchangeable effector locus, as indicated by the presence of *queA* and *tRNA_leu_*, which delineate the boundary of this pathogenicity island (Supplementary Figure S3). Several other accessory genes and putative virulence factors were identified in ICEs. Strain ZUM3985 carried *hopAW1* at a locus unique to the ICE found in this strain. It also carried a predicted calcium binding protein (1,691 aa) of the RTX toxin superfamily, which is a protein family commonly secreted via the type I secretion system (Linhartová et al., 2010). Likewise, a cassette of small peptides between 62 and 83 aa in length with a predicted *papB* domain, which is involved in the regulation of adhesin biosynthesis (Xia et al., 2000), was conserved in ICE of several strains including 13-140A, ZUM3584, 200-1, HS191, and SR121 (Figure 6).

**FIGURE 7:**
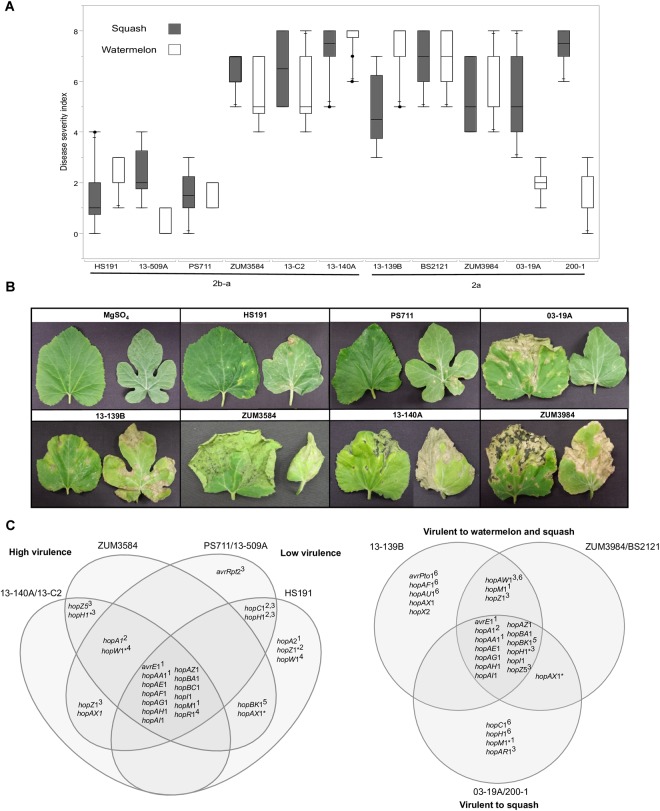
Correlation between T3SE profiles and pathogenicity of *P. syringae* strains to watermelon and squash. Pathogenicity analysis for representative cucurbit strains and the millet strain, HS191, from phylogroups 2a and 2b-a. Mean severity rating from 0 to 8, taken 14 days after inoculation from two independent growth room experiments (*n* = 10) **(A)**. Representative bacterial spot symptoms on squash and watermelon, respectively **(B)**. Comparison of effector repertoires among strains used in pathogenicity testing for phylogroups 2b-a (left) and 2a (right). An asterisk indicates a predicted disruption in the coding sequence and the superscript indicates the genomic regions of the effector gene in the genome assemblies: ^1^conserved effector locus, ^2^exchangeable effector locus, ^3^integrative and conjugative element locus, ^4^other genomic island, ^5^phage region, ^6^plasmid. An effector gene with multiple superscripts indicates a T3SE was acquired through different sources among strains **(C)**.

Analysis with plasmidSPAdes provided no evidence of plasmid sequences in phylogroup 2b-a cucurbit strains, whereas several were assembled among strains within phylogroup 2a. These results were consistent with the size of genome assemblies, which averaged 5.92 and 6.23 Mb for the 2b-a and 2a cucurbit strains, respectively (Table 1). A 52.42 Kb contig was assembled in strains 200-1 and 03-19A which displayed 93% nucleotide identity with 66% query coverage to the complete plasmid sequence from strain HS191 (NZ_CP006257.1). This plasmid carried the effectors *hopC1* and *hopH1*, which marked the second copy of *hopH1* for these two strains, in addition to the disrupted allele present in the ICE. A putative virulence plasmid of 16.51 Kb in size was assembled in strains 13-429 and 13-139B which harbored four accessory effector genes including *hopAU1*, *hopAW1*, *hopAF1*, and *avrPto*. The top hit for this contig in the NCBI complete plasmid database was plasmid PP3 (NZ_LT963405.1) of *P. syringae* pv. *avii* strain CFBP3846 and displayed only distant homology (95% nucleotide identity and 39% query coverage). Strains BS2121 and ZUM3984 also carried two putative plasmids, for which the top BLAST hits included the complete plasmids pCC1557 (NZ_CP007015.1) and pB13-200A (NZ_CP019872.1). However, no apparent virulence factors were identified in these contigs. A summary of the origin of T3SEs among representative cucurbit strains is presented in Figure 7C and a compiled list of putative plasmid sequences and BLAST results are available in Supplementary Table S1.

### Correlation Between T3SE Repertoires and Pathogenicity

The results from two independent pathogenicity experiments produced similar results and showed significant differences in disease severity between the treatments (*P* < 0.0001). Phylogroup 2b-a strains 13-140A, 13-C2, and ZUM3854 induced expanding lesions and foliar blighting on watermelon and squash, with mean severity ratings ranging from 5.60 to 7.50 among the three strains. In contrast, the squash strains 13-509A and PS711 produced only superficial lesions on both hosts and mean severity ratings that did not significantly differ from that of the millet strain, HS191 (mean severity ratings ranging from 0.70 to 2.40). Two effector pairs differentiated the virulent from weakly virulent strains within this group and included *hopZ*5 and the disrupted copy of *hopH1* among the virulent strains, while the weakly virulent strains carried *hopC1* and an intact copy of *hopH1*. Within phylogroup 2a, no significant differences in disease severity were observed among strains BS2121, ZUM3984, and 13-139B, which produced moderate to high levels of disease severity on both hosts. While strains 03-19A and 200-1 also induced expanding lesions and foliar blighting on squash (between 5.00 and 7.00 mean severity), they were weakly virulent on watermelon (between 1.40 and 2.00 mean severity). Four T3SEs were unique to the squash limited strains of phylogroup 2a and included *hopC1*, an intact copy of *hopH1*, *hopAR1*, and *hopM1* disrupted by a frameshift mutation. Likewise, *hopAW1*, *hopZ1*, and an intact *hopM1* allele were unique effectors common among the strains virulent to both watermelon and squash (Figure 7).

## Discussion

Bacterial leaf spot of watermelon, cantaloupe, and squash, caused by *P. syringae* (referring to *sensu stricto* unless otherwise stated), is a sporadic plant disease and often attributed to contaminated seed. Here, we provide the first genomic insights into the *P. syringae* strains associated with these three cucurbit hosts, with a focus on three apparently clonal lineages located in phylogroups 2a and 2b. We sequenced the genomes of six strains that were isolated from diseased cucurbits grown in the United States, Europe, and China over the period of a more than a decade, as well as eight strains that were isolated from watermelon and squash grown in six different Florida counties during the 2013 and 2014 seasons (Table 1). Reconstruction of the *P. syringae* core genome revealed that the cucurbit-associated lineages of phylogroup 2b formed a novel phylogenetic group, designated here as phylogroup 2b-a (Figure 1), which emerged through genome-wide homologous recombination between phylogroups 2a and 2b (Figure 2). While the majority of this group consisted of a single clonal lineage isolated from plants grown in the United States, France, and Serbia, strains ZUM3584 and HS191 were more distantly related and displayed variation in the distribution of recombinant loci that were dispersed across the core genome (Figure 1, 2). As the overall proportion of recombinant sequences was similar among all 2b-a lineages (ranging from 27.98 to 30.54% of the core genome), the distribution of recent recombination events, rather than the quantity, was the primary factor driving diversification within this group. These observations were supported by the recombination filtered core-genome phylogeny, which showed phylogroup 2b-a as a branch within phylogroup 2b, rather than as a distinct group, and more recently diverged as compared to the unfiltered core-genome phylogeny (Figure 2B,C). In contrast to the 2b-a strains, the *P. syringae* strains isolated from watermelon, cantaloupe, and squash in phylogroup 2a were nested among the lineages of other strains isolated from corn, wheat, switchgrass roots, and various tree species (Figure 1).

Unexpectedly, another example of genome-wide homologous recombination was inferred in our analysis among the *P. syringae* pv. *avellanae* (*Pav*) lineages of phylogroup Pav, whereby an estimated 40.09% of the core genome was acquired through ancestral recombination events (Supplementary Figure S1). These results were particularly interesting as the four *Pav* genomes analyzed were not clonal and displayed levels of sequence divergence equivalent to that of other *P. syringae* phylogroups (Table 3), suggesting that the fixation of recombinant alleles across phylogroup Pav (i.e., inference of ancestral recombination) was not an artifact of small sample size. These results were striking given the minimal impact of recombination on the evolution of the primary *P. syringae* phylogroups, which displayed an average of 95% pairwise identity between groups and were at the edge of the proposed species delimitation boundary for prokaryotes (Table 3). Both the recent and ancestral recombination profiles suggested admixture between phylogroups Pav and 2b (Figure 2A and Supplementary Figure S1). However, because phylogroup Pav did not display levels of pairwise identity intermediate of two different phylogroups, as was observed for 2b-a (Table 3), this could indicate that recombination has occurred between a currently unsampled *P. syringae* population. Taken together, these observations indicate that *P. syringae* phylogroups may inhabit overlapping environmental niches and are consistent with that of an epidemic population structure.

The link between macro-scale recombination events and the emergence of hybrid, epidemic-clones has been well documented among human and animal bacterial pathogens (Spoor et al., 2015). As such, genome-wide recombination events are associated with niche adaptation and remodeling of the host-pathogen interaction. Functional analysis of the recent recombination events that led to the emergence of phylogroup 2b-a painted a similar picture. Although a strong linear relationship between the COG category compositions suggested an overall proportional change among the general functional groups (Figure 3A), analysis utilizing the KEGG BRITE database revealed that pathways involved in the ATP-dependent transport of amino acids and other organic compounds, bacterial motility, and secretion systems were enriched for recombinant genes (Figure 3B). Interestingly, most of the specific pathways found within these functional groups were similarly affected by recombination in the millet pathogen, *P. syringae* pv. *syringae* HS191, except for those genes encoding for the type 3 secretion system, otherwise known as the *hrp/hrc* pathogenicity island. A phylogenetic analysis confirmed that the recombinant *hrp/hrc* genes shared a common evolutionary history with that of phylogroup 2a (Figure 4) and therefore, may be a significant factor contributing to the convergent pathogenicity of 2a and 2b-a lineages to cucurbit hosts. This finding was reminiscent of bacterial etiolation and decline of creeping bentgrass, for which the host-specificity of two phylogenetically distinct *Acidovorax avenae* populations was associated with three ancestral recombination events affecting the *hrp/hrc* gene cluster (Zeng et al., 2017).

In addition to genome-wide recombination, we found evidence for the remodeling of T3SE repertoires of phylogroup 2b-a. This remodeling was marked by the acquisition of *hopA1*, which was carried exclusively by most phylogroup 2a lineages within *P. syringae*, including those isolated from cucurbit hosts. The *hopA1* and *shcA* genes, which form an effector-chaperone complex, were the only two genes occupying the exchangeable effector locus of phylogroup 2b-a lineages (except for the millet strain HS191 which carried *hopA2*), suggesting that this effector was recently acquired. Previous studies have demonstrated that strains of *P. syringae sensu stricto* carry fewer T3SEs than other *Pseudomonas* spp. and this was associated with the production of broad host-range toxins such as syringomycin (Baltrus et al., 2011; Hulin et al., 2018). Although, we found evidence for the syringomycin, syringopeptin, and syringolin biosynthetic clusters in the genome assemblies (Supplementary Figure S2), on average, phylogroup 2b-a and 2a cucurbit strains carried more T3SEs (between 17 and 21 potentially functional or disrupted effector genes) than other *P. syringae* lineages (Figure 5). Most of these accessory T3SEs were acquired through independent mechanisms of HGT. These included an ICE, present in both 2a and 2b-a lineages (Figure 6), as well as two different plasmids harboring effector genes among phylogroup 2a lineages (Supplementary Table S1). Interestingly, a positive correlation between the proportion of recent recombination in the core genome and the number of T3SEs was observed in other *P. syringae* strains, including *P. cerasi* 58^T^, *P. syringae* pv. *papulans* CFBP1754^PT^, and *P. syringae* pv. *pisi* PP1 and highlights the interplay of homologous recombination and HGT in pathogen emergence (Figure 2, 5).

Despite the open pan-genome exhibited by *P. syringae* (Nowell et al., 2014), pan-genome association analysis identified only a handful of orthologous groups that were significantly associated (Bonferroni *p* ≤ 10^−5^) with both the 2a and 2b-a cucurbit strains (Table 4). Four of the seven significantly associated genes included previously described virulence factors such as the T3SE *hopZ5*, the *hopA1*/*shcA* effector-chaperone complex, and the type VI secreted effector *vgrG*, suggesting a role suggesting a role in pathoadaptation. The effector *hopZ5* was among several accessory T3SEs acquired through the ICE (Figure 6) and was the only virulence factor which distinguished the cucurbit-associated lineages from related pathogens of various plant species (Table 4). Interestingly, *hopZ5* was also among the few virulence factors acquired by the pandemic strains of *P. syringae* pv. *actinidiae* (*Psa*) biovar 3 (which may be considered a species distinct of *P. syringae sensu stricto*) and to date, has only been recorded in the genomes of *Pseudomonas* spp. associated with diseases of woody hosts (McCann et al., 2013; Nowell et al., 2016). Although *hopZ5* was not linked to the ICE described in *Psa* biovar 3, it was curious to find that many of the core ICE genes associated with the horizontal transfer of this effector among the cucurbit strains shared a recent evolutionary history with the ICE carried by *Psa* strain SR121 (Figure 6).

The acquisition of novel virulence factors through HGT is commonly attributed to changes in bacterial phenotypes. Hence, it was striking to note the contrasting pathogenicity among the cucurbit strains (Figure 7). As these differences in pathogenicity were observed among strains of the same clonal lineage, this indicated that components of the accessory genome, rather than the underlying genetic background was likely the key factor influencing these phenotypes. We found that two phylogroup 2b-a strains collected in Florida and Serbia (13-509A and PS711, respectively) were both weak pathogens of watermelon and squash and produced superficial lesions like that of the millet strain HS191 (Figure 7A,B). Interestingly, 13-509A and PS711 also carried effector repertoires more like that of the millet strain than other virulent 2b-a strains isolated from cucurbits (Figure 5). This difference was primarily accounted for by the presence of *hopC1*, in place of *hopZ5* in the ICE locus, while *hopC1* was carried in the exchangeable effector locus of strain HS191 (Figure 7C). Both *hopZ5* and *hopC1* were adjacent to a second T3SE, *hopH1* (Figure 6). However, the *hopH1* allele present in the virulent cucurbit strains carried a point deletion, rendering a frameshift mutation in the gene and was therefore not predicted to be translocated or expressed.

A similar negative association between the *hopC1* and *hopH1* effector pair and virulence was observed among phylogroup 2a strains 03-19A and 200-1. Both strains were weakly pathogenic to watermelon and carried *hopC1* and an intact copy of *hopH1* on a putative ∼52 Kb plasmid (Figure 7C). While the disruption of *hopH1* among highly virulent strains within phylogroups 2a and 2b-a may serve to avoid host recognition, we cannot discount the possibility that the disruption of this effector was an artifact due its horizontal transfer and linkage to *hopZ5*, rather than selection pressure. Furthermore, we observed that strains 03-19A and 200-1 induced severe foliar blighting on squash, rather than being weakly pathogenic in general (Figure 7A,B). This indicates a difference in the nature of the host-pathogen interaction and perhaps suggests induction of an effector triggered immunity in watermelon. These strains also carried *hopAR1* in the ICE locus (Figure 7C), which is an avirulence gene that has been well described in *P. syringae* pv. *phaseolicola* (Tsiamis et al., 2000) and is another candidate potentially limiting the host-range of these two stains to squash. Further analysis is required to determine whether the expression of any of the gene candidates identified here, including *hopC1*, *hopH1*, and *hopAR1* may serve as negative pathogenicity factors in cucurbits, and conversely, whether *hopZ5* promotes the virulence of these pathogens.

The widespread distribution of two genetically monomorphic *P. syringae* populations in association with multiple cucurbit hosts suggests a role for natural selection in maintaining these populations. Furthermore, the convergent acquisition of alleles through homologous recombination and multiple virulence factors through HGT among the cucurbit strains examined here may be interpreted as genomic signatures of host-adaption (Sheppard et al., 2018). Although the evolutionary processes inferred here mirrored those of other plant–pathogenic bacteria responsible for an array of emerging diseases, we do not know if these recombination events occurred upon colonization of cucurbit hosts or if these events happened prior to exposure to this ecological niche. Clues as to the answer of this question may found in the recombinant genome of the millet pathogen, *P. syringae* pv. *syringae* HS191, which lacked similar signatures in ecologically significant loci such as the *hrp*/*hrc* pathogenicity island and ICE locus. The significance of other numerous functional pathways affected by recent recombination events remains to be explored and provides further evidence for the emerging paradigm that plant–pathogen compatibility is not defined solely by T3SE repertoires but is likely a multifactorial process involving the acquisition/metabolism of plant derived nutrients, bacterial chemotaxis, and evasion of plant-innate immunity, among other processes (Jacques et al., 2016). Ultimately, this hypothesis will need to be tested through more extensive sampling of *P. syringae* strains from multiple environments coupled with functional analysis.

## Author Contributions

EN, ME, JJ, EG, and MP conceived the project. MP, CB, NZ, EN, and AO provided *P. syringae* strains. EN conducted the pathogenicity assays. ME prepared the genomic DNA. ME, ST, and JJ oversaw the sequencing experiments. ST and NP assembled the draft genomes. ME conducted the toxin analysis and EN performed all other computational analyses with support from JH-T and NP. ME submitted the genome sequences to NCBI-GenBank and JGI-IMG. EN wrote the manuscript and all authors provided a critical review of the paper.

## Conflict of Interest Statement

The authors declare that the research was conducted in the absence of any commercial or financial relationships that could be construed as a potential conflict of interest.

## Footnotes

1 https://github.com/vsbuffalo/scythe

2 https://github.com/widdowquinn/pyani

3 http://tree.bio.ed.ac.uk/software/figtree/

4 http://www.pseudomonas-syringae.org/

5 https://bitbucket.org/nsegata/hclust2
