# Corrigendum: Inference of Convergent Gene Acquisition Among *Pseudomonas syringae* Strains Isolated From Watermelon, Cantaloupe, and Squash

**DOI:** 10.3389/fmicb.2019.00963

**Published:** 2019-05-03

**Authors:** Eric A. Newberry, Mohamed Ebrahim, Sujan Timilsina, Nevena Zlatković, Aleksa Obradović, Carolee T. Bull, Erica M. Goss, Jose C. Huguet-Tapia, Mathews L. Paret, Jeffrey B. Jones, Neha Potnis

**Affiliations:** ^1^Department of Entomology and Plant Pathology, Auburn University, Auburn, AL, United States; ^2^Department of Plant Pathology, North Florida Research and Education Center, University of Florida, Quincy, FL, United States; ^3^Department of Plant Pathology, University of Florida, Gainesville, FL, United States; ^4^Department of Plant Pathology, Faculty of Agriculture, Ain Shams University, Cairo, Egypt; ^5^Faculty of Agriculture, University of Belgrade, Belgrade, Serbia; ^6^Department of Plant Pathology and Environmental Microbiology, Pennsylvania State University, State College, PA, United States; ^7^Emerging Pathogens Institute, University of Florida, Gainesville, FL, United States

**Keywords:** horizontal gene transfer, homologous recombination, pathogen emergence, *Pseudomonas syringae sensu stricto*, cucurbits

In the original article, we neglected to acknowledge the University of Florida Open Access Publishing Fund in supporting the publication of this manuscript. The authors apologize for this error and state that this does not change the scientific conclusions of the article in any way. The original article has been updated.

