# Supplementary material for: Inference of Convergent Gene Acquisition Among Pseudomonas syringae Strains Isolated From Watermelon, Cantaloupe, and Squash
Source: Front Microbiol. 2019 Feb 19;10:270. doi: 10.3389/fmicb.2019.00270 (PMC6390507; doi:10.3389/fmicb.2019.00270)
Supplement: Supplementary file 1 [file Data_Sheet_1.docx]

| **Supplementary Table S1. Assembly statistics and BLAST results of plasmids detected using plasmidSpades.** | | | | | | | |
| --- | --- | --- | --- | --- | --- | --- | --- |
| **Strain** | **Putative Plasmids** | **Size (bp)** | **Best Hit** | **Identity (%)** | **Converage (%)** | **E-value** | **Effector genes** |
| 03-19A | NODE_1_length_52420_cov_7.20727_component_0 | 52420 | Pseudomonas syringae pv. syringae HS191 plasmid, complete sequence (NZ_CP006257.1) | 93 | 66 | 0 | hopC1,hopH1 |
|  | NODE_3_length_2149_cov_6.71068_component_0 | 2149 | Pseudomonas syringae pv. maculicola str. M6 plasmid pFKN, complete sequence (NC_002759.1) | 96 | 75 | 0 | NA |
| 200-1 | NODE_1_length_52421_cov_7.9271_component_0 | 52421 | Pseudomonas syringae pv. syringae HS191 plasmid, complete sequence (NZ_CP006257.1) | 93 | 66 | 0 | hopC1,hopH1 |
|  | NODE_3_length_2149_cov_10.0742_component_0 | 2149 | Pseudomonas syringae pv. maculicola str. M6 plasmid pFKN, complete sequence (NC_002759.1) | 96 | 75 | 0 | NA |
| BS2121 | NODE_1_length_73409_cov_5.9803_component_0 | 73409 | Pseudomonas syringae CC1557 plasmid pCC1557, complete sequence (NZ_CP007015.1) | 93 | 50 | 0 | NA |
|  | NODE_2_length_1447_cov_8.84091_component_0 | 1447 | Pseudomonas syringae pv. tomato strain B13-200 plasmid pB13-200A, complete sequence (NZ_CP019872.1) | 99 | 83 | 0 | NA |
| ZUM3984 | NODE_2_length_22679_cov_4.66894_component_1 | 22679 | Pseudomonas syringae CC1557 plasmid pCC1557, complete sequence (NZ_CP007015.1) | 93 | 73 | 0 | NA |
|  | NODE_4_length_5701_cov_9.77682_component_0 | 5701 | Pseudomonas syringae pv. tomato str. DC3000 plasmid pDC3000A, complete sequence (NC_004633.1) | 95 | 57 | 0 | NA |
| 13-139B | NODE_1_length_16509_cov_6.29448_component_0 | 16509 | Pseudomonas syringae pv. avii isolate CFBP3846 genome assembly, plasmid: PP3 (NZ_LT963405.1) | 95 | 39 | 0 | hopAU1,hopAW1,hopAF1,avrPto |
|  | NODE_2_length_14660_cov_6.95043_component_0 | 14660 | Pseudomonas savastanoi pv. phaseolicola 1448A large plasmid, complete sequence (NC_007274.1) | 99 | 47 | 0 | NA |
|  | NODE_4_length_3283_cov_12.6229_component_0 | 3283 | Pseudomonas savastanoi NCPPB 3335 native plasmid pPsv48C complete sequence (NC_019292.1) | 99 | 50 | 0 | NA |
| 13-429 | NODE_3_length_16509_cov_9.28342_component_2 | 16509 | Pseudomonas syringae pv. avii isolate CFBP3846 genome assembly, plasmid: PP3 (NZ_LT963405.1) | 95 | 39 | 0 | hopAU1,hopAW1,hopAF1,avrPto |
|  | NODE_4_length_14660_cov_10.3435_component_2 | 14660 | Pseudomonas savastanoi pv. phaseolicola 1448A large plasmid, complete sequence (NC_007274.1) | 99 | 47 | 0 | NA |
|  | NODE_7_length_3283_cov_17.115_component_2 | 3283 | Pseudomonas savastanoi NCPPB 3335 native plasmid pPsv48C complete sequence (NC_019292.1 ) | 99 | 50 | 0 | NA |

**
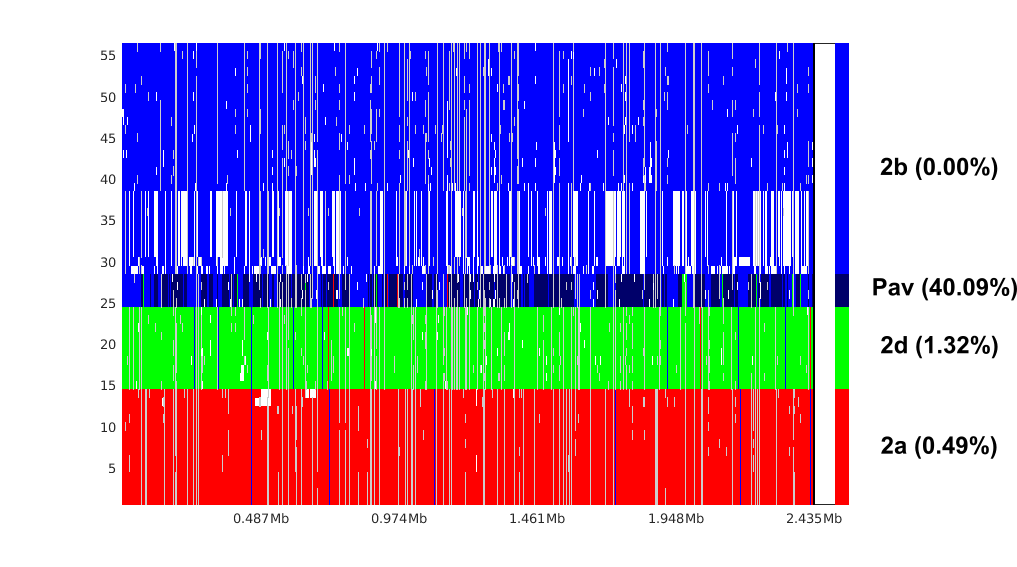
Supplementary figure S1.** Ancestral recombination events across the events across the *P. syringae* phylogroup 2 core genome as predicted by fastGEAR. The lineage predictions are color coded and the proportion of core genome affected by ancestral recombination is shown in parentheses.


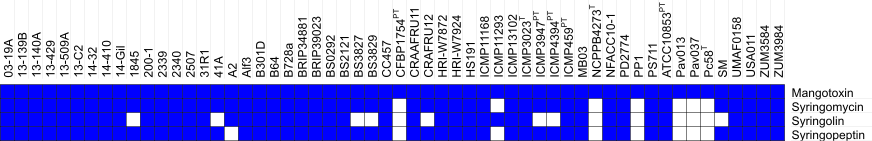
**Supplementary figure S2**. Distribution of phytotoxin biosynthetic clusters across *P. syringae* phylogroup 2. Blue squares indicate the presence of the completed biosynthetic pathway and white squares show incomplete or absent pathways.


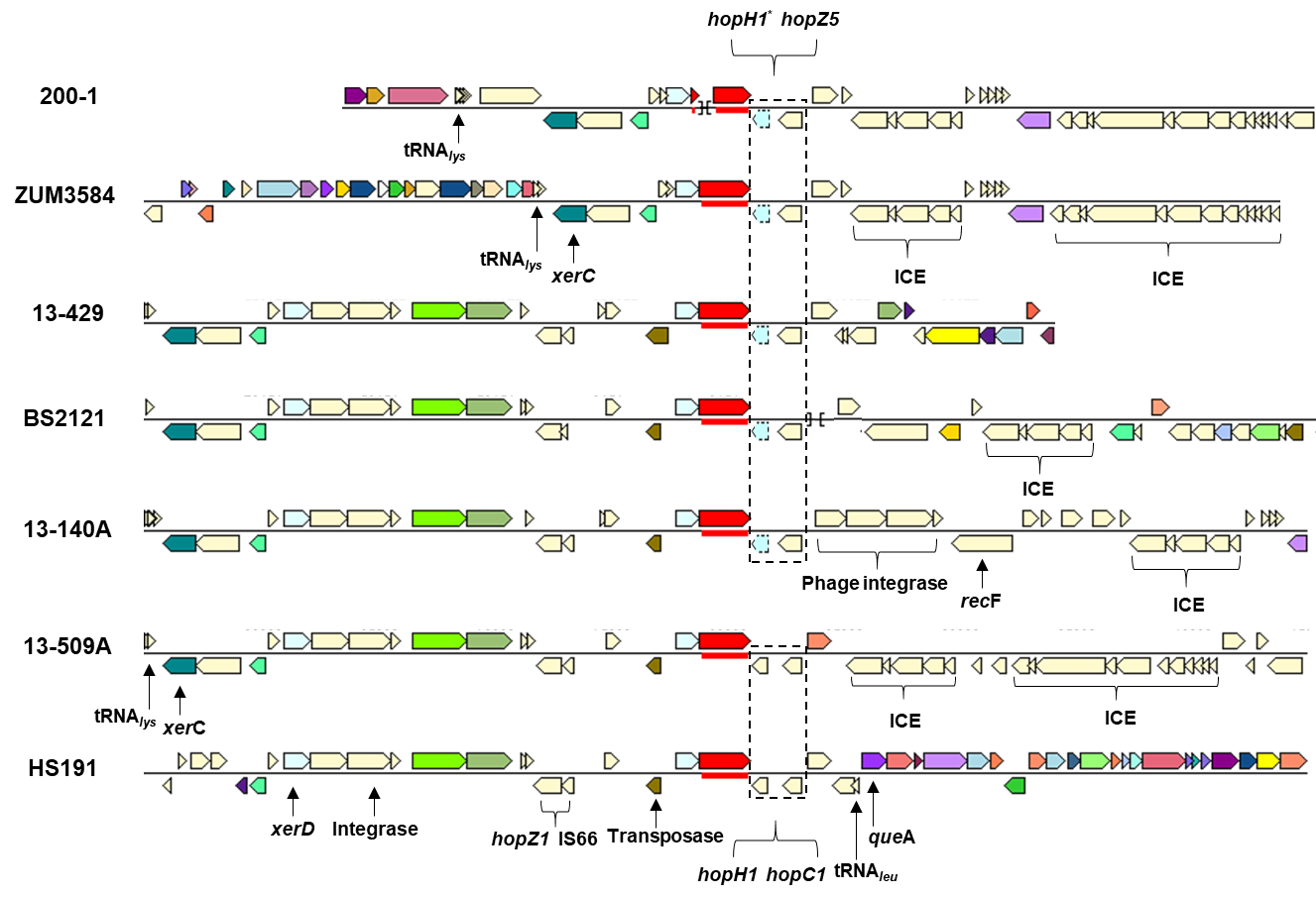
**Supplementary figure S3**. Comparison of the *hopZ5*/*hopC1* locus among representative cucurbit strains and the millet strain, HS191. Boxes of the same color indicate orthologous genes and the predicted phage integrase linked to *hopZ5* and *hopC1* is underlined in red. An asterisk indicates a predicted pseudogene and ICE denotes predicted integrative and conjugative element associated gene cassettes.
